# Food Advertising Literacy Training Reduces the Importance of Taste in Children’s Food Decision-Making: A Pilot Study

**DOI:** 10.3389/fpsyg.2018.01293

**Published:** 2018-07-27

**Authors:** Oh-Ryeong Ha, Haley Killian, Jared M. Bruce, Seung-Lark Lim, Amanda S. Bruce

**Affiliations:** ^1^Department of Psychology, University of Missouri–Kansas City, Kansas City, MO, United States; ^2^Department of Biomedical and Health Informatics, University of Missouri–Kansas City, Kansas City, MO, United States; ^3^Department of Pediatrics, University of Kansas Medical Center, Kansas City, KS, United States; ^4^Center for Children’s Healthy Lifestyles and Nutrition, Children’s Mercy Hospital, Kansas City, MO, United States

**Keywords:** decision-making, food choices, advertising literacy, food advertising, youth, obesity

## Abstract

Television food advertising influences children’s food choices. The attribute of “taste” drives children’s food choices, and exposure to food commercials can increase the importance of “taste” when children make food decisions. The current pilot study explored whether food advertising literacy training influences children’s food choices. In particular, whether the training would change the way children weigh the importance of taste attributes in their food decisions. Thirty-nine children ages 8–13 were recruited. Twenty-three of those children had four sessions of food advertising literacy training (1 week): children watched four videos of food commercials embedded with factual narratives (i.e., building cognitive defenses; e.g., “commercials want to sell products”) and evaluative narratives (i.e., changing affective responses toward commercials; e.g., “these foods don’t make you happy”). The first and last sessions were held in the laboratory, and the second and third sessions were at home. During the training, children were encouraged to think aloud while watching commercials and provided narratives to encourage active information processing. At baseline and post-training, children made binary eating choices for 60 foods and rated each food item on health and taste. We fitted linear regression models to examine whether taste and health attributes predicted unique variance in each child’s food choices. The results showed that taste attributes in children’s food choices was significantly decreased after completing the training. This finding suggested that improving food advertising literacy could be helpful for reducing the influence of taste attributes in the food decision-making process. Also, the cognitive literacy training increased children’s critical thoughts toward commercials during thinking aloud. These findings suggest that food advertising literacy training was helpful for reducing the importance of “taste” in children’s food decisions. In contrast, 16 children in the control condition (i.e., watching four videos of food commercials without narratives in 1 week) did not show any significant change in their food choices. Future research should investigate the utility of food advertising literacy training for the promotion of healthy eating and the prevention of childhood obesity.

## Introduction

Companies spend billions of dollars each year to advertise products to youth (Packaged Facts, 2016). If a company can hook youth early by achieving brand recognition and brand preference, they have created a loyal customer for life. By the age of two, 90% of children are exposed to television media daily (Zimmerman et al., 2007). Published estimates show children are bombarded by at least 5,500 advertisements per year (Federal Trade Commission and Department of Health and Human Services, 2006) and up to 98% of these advertised food/drink are high in fat, sugar, and salt (Powell et al., 2007).

Advertising exerts a powerful influence on youth, as children strongly prefer advertised foods to non-advertised foods (Coon and Tucker, 2002; McGale et al., 2016). Increased exposure to advertising directly impacts the number of attempts children make to influence their parents’ food purchases (Coon and Tucker, 2002). Even when foods are identical save for packaging, children express significant preference for the taste of branded foods over unbranded foods (Robinson et al., 2007). Television food advertising influences children’s food choices and food consumption (Boyland et al., 2012, 2016; Boyland and Halford, 2013).

These research studies suggest food advertising exerts a powerful, potentially harmful influence on children’s eating behaviors. Evidence suggests this practice is contributing to the increasing rate of childhood obesity (Cairns et al., 2013; Kelly et al., 2015). Obese and healthy weight children respond differently to marketing and one study showed that overweight children consume significantly more calories of advertised foods than their healthy weight peers (Forman et al., 2009). As a result, in the past decade, researchers and policy groups are questioning the ethics of marketing unhealthy foods toward children (Harris and Kalnova, 2018).

Many factors drive food choices from how rewarding they are (pleasant taste) to social influences (peers, marketing) (Bruce et al., 2015). When children make food choices, the attribute of taste is usually most important (Ha et al., 2016; Lim et al., 2016). Our previous work shows that exposure to food commercials can increase the importance of “taste” in children’s food decisions (Bruce et al., 2016).

Experts have begun to develop interventions designed to “inoculate” youth against the effects of marketing. Still, there are relatively few of these studies, and it is not established yet what approaches would work to counter advertising in youth. For example, one study of over 1000 elementary students showed that front-of-pack nutritional labels increased the desire for unhealthy foods in children (Dixon et al., 2014). Nevertheless, a few studies have shown that advertising literacy can be an effective counter advertising strategy. One study showed that an intervention focused on food advertising literacy increased nutritional knowledge in children (Nelson and Kehr, 2016). A recent published intervention emphasized parent–child discussion about food and nutritional labels (Austin et al., 2018). Some behavioral interventions incorporate “media literacy” or “health literacy” training as part of a randomized controlled trial aimed at reducing consumption of sugar-sweetened beverages (Zoellner et al., 2016).

The current study examined whether a brief, food advertising literacy training is associated with changes in children’s food decision-making. We hypothesized that the relative importance of “taste” decreases in food choices after completing four sessions of food advertising literacy training. We also hypothesized that the food advertising literacy training would enhance children’s healthier food choices, and critical thought toward commercials.

## Materials and Methods

### Participants

Thirty-nine healthy children (19 girls, 20 boys) ages 8–13 years (*M* = 10.34 years, *SD* = 1.36) with normal or corrected-to-normal vision participated. All participants were recruited from the Kansas City metropolitan area and spoke English as their first language. The racial background of the participants was 13 Caucasian (33%), 1 African American (0.3%), 4 multiracial (1%), and 21 Asian (54%). The mean body mass index (BMI) was 19.28 (kg/m^2^; *SD* = 4.23, range 13.2–34.2), and the mean BMI-percentile-for-age was 62.76 (*SD* = 29.28, range: 1.0–99.5). Based on the BMI-percentile-for-age, children were categorized as underweight (*n* = 2; 5%), healthy weight (*n* = 24; 52%), overweight (*n* = 6; 15%), and obese (*n* = 7; 18%). Two participants were excluded from data analyses because they failed to complete the experiment. Among 39 children, 23 children had the advertising literacy training sessions, and 16 children were participated in the control condition. When we compared children in the training and control conditions, there were no significant age and BMI-percentile-for-age differences [*t*(37) = 0.011, *p* = 0.992; *t*(37) = 0.34, *p* = 0.736]. This study was approved by the Institutional Review Board at the University of Missouri-Kansas City and the Human Subjects Committee at the University of Kansas Medical Center.

### Materials

#### Food Advertising Literacy Training

The advertising literacy training was modeled after a program designed to expose children to factual and evaluative narratives that help them critically evaluate advertising (Buijzen, 2007), researchers targeted children’s susceptibility to commercials by stimulating their advertising knowledge and skepticism (factual narratives), and by influencing their attitudes toward commercials negatively (evaluative narratives) at one session. We aimed to take it a step further to examine what the training did to food choices.

**FIGURE 1:**
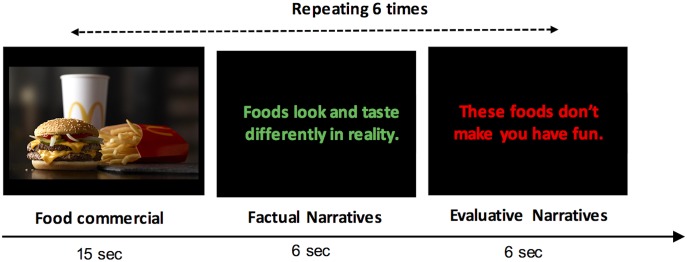
Procedure for the food advertising literacy training. Food advertising literacy information was delivered via video. Each video consisted of 6 commercials and 12 factual and evaluative narratives. The session began with a food commercial that was followed by two factual or evaluative narratives. Each narrative sentence in a different color moved side-to-side and was accompanied by a female voice. Two sets of video stimuli were used alternately across four sessions.

The food advertising literacy training was delivered via video (**Figure 1**). Using 12 television food commercials and 12 factual and evaluative narratives, two training videos were created. Each training video included six television food commercials advertising Applebee’s^®^, Chili’s Grill & Bar^®^, Denny’s^®^, McDonald’s^®^, Subway^®^, and Wendy’s^®^ brands. The commercials were used by Gearhardt et al. (2014) and were also used in our previous study examining brain activity and the influence of food commercials on children’s food choices (Bruce et al., 2016). For delivering the food advertising literacy, 12 narratives were embedded in the training videos: five factual narratives for providing information about advertising knowledge and skepticism (e.g., these commercials are intended to sell) and seven evaluative narratives for changing affective responses toward commercials (e.g., these foods don’t make you happy) (**Table 1**). All 12 narratives including both factual and evaluative narratives were delivered to children in one training video. Each commercial was followed by two narratives presented one by one. Each narrative statement was shown in colored text on a black background that moved side-to-side on the screen to maintain children’s attention and was accompanied by a female voice reading the narrative. Each commercial lasted approximately 15 s and two narratives were presented for approximately 12 s (6 s/narrative). The order of food commercials and narratives were randomized for each video stimulus before creating the training videos. Each child watched one of two training videos per session, and the order of the two training videos was counterbalanced across participants (e.g., video 1, video 2, video 1, video 2).

**Table 1:** Factual and evaluative narratives for food advertising literacy training.

Factual narratives	Evaluative narratives
(1) Foods look and taste differently in reality.	(1) These foods don’t make you have fun.
(2) The advertisers want you to go and eat these foods.	(2) Those foods are disgusting.
(3) These commercials are intended to sell.	(3) People in these commercials aren’t cool.
(4) The advertisers are trying to trick you.	(4) These foods don’t make you happy.
(5) These commercials aren’t telling the truth.	(5) These foods are bad for you.
	(6) Those foods are not delicious.
	(7) Those foods are so unhealthy.

#### Control Condition

The control video was identical to the training video, but no narratives for enhancing advertising literacy were embedded. The same 12 food commercials used in the training videos were used to create two control videos. Each commercial was followed by a blank screen with no text or sound for 12 s. Similar to the training condition, each child watched one of two control videos per session, and the order of two control videos was counterbalanced across participants.

#### Food Rating and Choice Tasks

**FIGURE 2:**
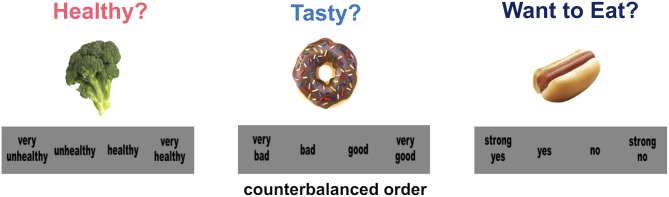
Examples of food health rating, taste rating, and food choice tasks.

See **Figure 2** for the examples of food rating and choice tasks. Prior to beginning the behavioral tasks, the research staff confirmed that children could recognize all 60 food items. Color food images included 30 healthy food items such as vegetables, fruits, and beans and 30 unhealthy food items such as fried foods, sweet desserts, and processed meats. In the food rating tasks, children rated food items for health attributes (unhealthy to healthy: “very unhealthy” to “very healthy”) and taste attributes (bad to good: “very bad” to “very good”) using a four-point scale. The order of rating options was counter-balanced so that some participants rated food items using the opposite order (e.g., “very unhealthy” to “very healthy” or “very healthy” to “very unhealthy”). The rating order was applied consistently for each participant across all behavioral tasks. Health and taste attributes were measured in separate rating tasks, and the test order of health and taste attributes ratings was counterbalanced across participants. An initial instruction display labeled each task distinctively. Children were instructed to rate health attributes regardless of taste attributes and taste attributes regardless of health attributes. A high resolution, color food image (72 dpi; 300 × 300 pixels) was presented on a white background on a laptop monitor in a randomized order. The rating scale was displayed below a food image in black text color within a gray box. A food image stayed on the screen until a child made a response using buttons on the keyboard. A chosen rating option was displayed in a yellow text color to confirm children’s response. A fixation cross was presented for 1 s between trials. In the food choice task that followed, children were asked to choose whether or not they would eat each food item using a four-point rating scale (“strong no” to “strong yes”). To ensure children’s motivation for making real eating choices, they were told they would receive one of food item they chose to eat in the food choice task after the completion of the session. Presentation software (version 19; Neurobehavioral Systems, Berkeley, CA, United States) controlled the stimulus presentation and response collection.

#### Think-Aloud

While watching the food advertising literacy training video, children were encouraged to say what they were thinking out loud to enhance active information processing (Rozendaal et al., 2012). Children’s spoken words were recorded during commercials and narratives using a voice recorder application on an experimenter’s smart phone. Children in the control condition were also encouraged to speak out loud what they were thinking while watching the control video.

##### Coding

Children’s spoken statements were categorized using a coding scheme into four dimensions (Table 1 in Rozendaal et al., 2012): (1) Relevance of thought, whether expressed thoughts were related to the commercial (relevant) or unrelated (irrelevant); (2) origin of thought, whether statements were merely describing the visual or verbal contents of the commercial (message-originated) or delivering children’s thoughts (recipient-generated); (3) nature of thought, whether thoughts were related to beliefs about the commercial (cognitive) or affective responses toward the commercial (affective); and (4) polarity of thought, whether thoughts were negative, neutral, or positive toward the commercial. Additionally, whether children’s thought expressed understanding of advertising intents and tactics (understanding) was coded. Two research staffs coded children’s statements independently.

##### Cognitive and affective critical thoughts

After all statements were coded, cognitive critical thoughts and affective critical thoughts were computed. Only relevant and recipient-generated thoughts were included in the computation, and thoughts were categorized as positive cognitive (e.g., “That was definitely told the truth”), negative cognitive (e.g., “This doesn’t look healthy”), positive affective (e.g., “This breakfast looks good”), and negative affective (e.g., “Disgusting”). Cognitive critical thoughts were computed by subtracting the number of positive cognitive thoughts from the sum of negative cognitive thoughts and understanding. Cognitive critical thoughts indicated a child understood the intent and tactics of the commercial and processed commercial information more critically. Affective critical thoughts were calculated by subtracting the number of positive affective thoughts from the number of negative affective thoughts. Affective critical thoughts indicated that a child expressed negative attitudes toward the commercial or advertised foods.

#### Advertising Literacy Scale for Children

The modified Advertising Literacy Scale for Children (ALS-c) (Rozendaal et al., 2016) was used. This scale was intended to measure children’s understanding of advertising intent and tactics and their critical attitude toward advertising. The original ALS-c was consisted of a 16-item Conceptual Advertising Literacy Scale for Children (CALS-c) and a 9-item Attitudinal Advertising Literacy Scale (AALS-c). Because these scales were designed to be completed after watching three commercials, we added six items to cover our six commercials.

### Procedure

**FIGURE 3:**
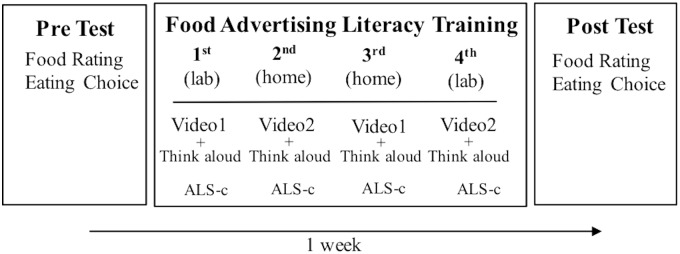
Food advertising literacy training timeline.

Over 1 week (*M* = 7.13 days, *SD* = 0.69), children had four sessions of food advertising literacy training (**Figure 3**). The first and last sessions were held in the laboratory, and the second and third sessions were held at home. The food rating and choice tasks were completed in the laboratory a total of two times, before and after completing the entire four sessions of food advertising literacy training, for measuring the training effect. Identical food ratings and choice tasks with same food images were used in pre- and post-training to measure changes in food ratings and choices after completing the training.

#### First Training Session in Laboratory

Children were asked to refrain from eating for 2 h prior to coming into the laboratory to ensure heightened motivation when they made food choices. To calculate BMI, children’s heights and weights were measured in light street clothing and stocking feet using a Befour MX805 scale. Participants completed an 11-point visual analog scale to report their current levels of hunger (*M* = 4.91, *SD* = 3.53) before the beginning of the food rating and choice tasks. Children completed food rating and choice tasks before getting any advertising literacy training to provide a baseline measure of food ratings and choices. Then children had the first food advertising literacy training, and completed the ALS-c. At the end of the first training session, research staff gave instructions to parents for two training sessions at home. Parents were sent home with two food advertising literacy training video files saved on a USB flash drive and copies of ALS-c.

#### Second and Third Training Sessions at Home

Children were asked to watch training videos on two different days before returning to the laboratory for the fourth session. Parents were asked to give think-aloud directions to children before watching the training videos. Parents recorded children’s vocalizations using a voice record application on their smart phones or using computer media players. Children completed the ALS-c after each training session.

#### Fourth Training Session in the Laboratory

Children came into the laboratory for the last training session a week after the initial session. This session began with viewing the food advertising literacy training video and completing the ALS-c. Children reported their hunger level using the visual analog scale before beginning the behavioral tasks (*M* = 4.16, *SD* = 3.37). After completing the last session of training, children completed the food rating and choice tasks to provide a training outcome measure of food ratings and choices.

#### Control Sessions

Similar to the training, children in the control condition had four control sessions over 1 week (*M* = 7.06 days, *SD* = 0.25). The first and last sessions were held in the laboratory, and the second and third sessions were held at home. At the first session, children reported their current level of hunger using the visual analog scale and completed the food rating and choice tasks (*M* = 5.73, *SD* = 2.49). Then children watched the control video and completed the ALS-c. Children watched control videos on two different days at home and completed the ALS-c after each control session. Children came back to the laboratory for the last control session after 1 one week. Children watched the control video and completed the ALS-c. Children reported current hunger level (*M* = 5.45, *SD* = 3.09), and then completed the food rating and choice tasks.

## Results

We modeled statistical analyses on our previous work demonstrating an increase of the value of taste attributes after watching food commercials (Bruce et al., 2016). To compute each child’s decision values of taste attributes and health attributes in food choices, we fitted a linear regression model of predicting food choices from taste and health ratings in each child. A beta coefficient of taste attributes represented the relative importance of the taste in food choices, and a beta coefficient of health attributes represented the relative importance of the healthiness in food choices at the individual level.

### Determinants of Food Choices

**FIGURE 4:**
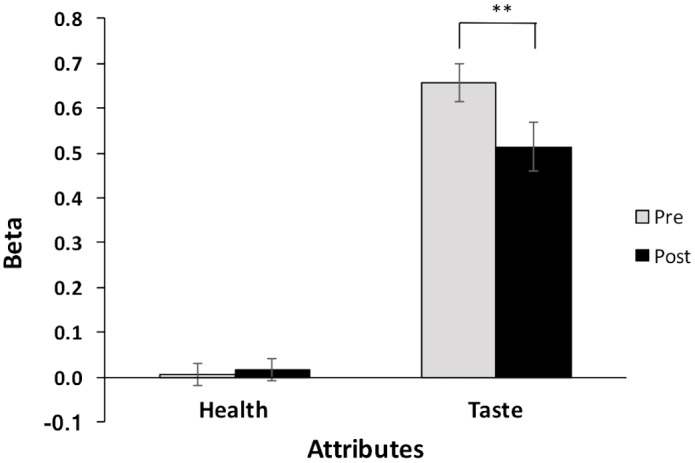
Mean beta coefficients of health and taste attributes at pre- and post-training. ^∗∗^*p* < 0.01.

We tested how children incorporated food taste and health attributes in their food decision-making. For group-level analyses, we conducted *t*-tests with regression beta coefficients of taste and health attributes for pre- and post-training separately. The results demonstrated that taste ratings significantly predicted children’s food choices before [mean β*_pre_* = 0.66, *t*(22) = 15.35, *p* < 0.0001, *d* = 3.2] and after completing the training [mean β*_post_* = 0.51, *t*(22) = 9.26, *p* < 0.0001, *d* = 1.93]. In contrast, health ratings did not significantly predict food choices at either time [mean β*_pre_* = 0.005, *t*(22) = 0.20, *p* = 0.85, *d* = 0.04; mean β*_post_* = 0.016, *t*(22) = 0.67, *p* = 0.51, *d* = 0.14] (**Figure 4**).

### The Food Advertising Literacy Training Effect

First, we examined if children’s food choices had changed before and after completing the advertising literacy training. A planned paired *t*-test on the food decision value measured by the food choice task did not show significant differences across training sessions [*M_pre_* = 3.07, *M_post_* = 3.01, *t*(22) = 1.41, *p* = 0.17, *d* = 0.30].

Next, to evaluate whether the importance of taste in food choices had changed after completing trainings at the group-level, we conducted a planned paired *t*-test with regression beta coefficients of taste attributes for pre-training and post-training. As we hypothesized, the relative importance (value) of taste attributes decreased significantly from pre- to post-training [*M_pre_* = 0.66, *M_post_* = 0.51, *t*(22) = 3.41, *p* = 0.003, *d* = 0.71]. Additionally, to examine whether the health importance had changed with trainings, we conducted a paired *t*-test with regression beta coefficients of health attributes for pre-training and post-training. However, the relative importance of health attributes was not changed significantly across pre- and post-trainings [*M_pre_* = 0.005, *M_post_* = 0.016, *t*(22) = -0.44, *p* = 0.666, *d* = 0.091]. These findings suggested that the training reduced importance of taste in food decisions, but did not influence the relative importance of health attributes in food decisions.

Then, we examined whether children’s advertising literacy measured by the ALS-c had increased. Planned paired *t*-tests did not show significant difference on conceptual advertising literacy [CALS-c: *M_pre_* = 68.83, *M_post_* = 73.00, *t*(22) = -1.80, *p* = 0.085, *d* = 0.38], attitudinal advertising literacy [AALS-c: *M_pre_* = 25.78, *M_post_* = 26.04, *t*(22) = -0.43, *p* = 0.673, *d* = 0.09], and total advertising literacy [ALS-c: *M_pre_* = 94.61, *M_post_* = 99.04, *t*(22) = -1.76, *p* = 0.092, *d* = 0.37] across training sessions.

Last, we examined if there was a change in children’s cognitive and affective critical thoughts verbalized while viewing the training video clips. Planned paired *t*-tests showed that cognitive critical thoughts, while watching commercials, significantly increased at the last training session compared to the first session [*M_first_* = 0.22, *M_last_* = 0.91, *t*(22) = 2.24, *p* = 0.036, *d* = 0.47], but affective critical thoughts did not [*M_first_* = -0.48, *M_last_* = -0.17, *t*(22) = 1.13, *p* = 0.173, *d* = 0.23].

### Control Condition

We examined the determinants of food choices, and the control session effect using the same statistical analyses described above. Similar to children in the training condition, taste ratings significantly predicted children’s food choices before [mean β*_pre_* = 0.68, *t*(15) = 14.79, *p* < 0.0001, *d* = 3.7] and after completing the control sessions [mean β*_post_* = 0.64, *t*(15) = 10.59, *p* < 0.0001, *d* = 2.65]. Health ratings did not significantly predict food choices at either time [mean β*_pre_* = 0.015, *t*(15) = 0.46, *p* = 0.652, *d* = 0.12; mean β*_post_* = 0.0009, *t*(15) = 0.02, *p* = 0.984, *d* = 0.005]. The food decision value measured by the food choice task did not show significant differences across control sessions [*M_pre_* = 2.77, *M_post_* = 2.77, *t*(15) = -0.08, *p* = 0.937, *d* = 0.02].

Most importantly, unlike the training condition, the relative importance of taste attributes was not decreased significantly from pre- to post-control sessions [*M_pre_* = 0.68, *M_post_* = 0.64, *t*(15) = 0.615, *p* = 0.548, *d* = 0.15]. And the relative importance of health attributes was not changed significantly across control sessions [*M_pre_* = 0.015, *M_post_* = 0.0009, *t*(15) = 0.354, *p* = 0.728, *d* = 0.09]. These findings suggested that the relative importance of taste attributes was decreased only in children in the training condition.

Similar to the training condition, conceptual advertising literacy [CALS-c: *M_pre_* = 69.19, *M_post_* = 71.13, *t*(15) = -0.90, *p* = 0.384, *d* = 0.22], attitudinal advertising literacy [AALS-c: *M_pre_* = 24.50, *M_post_* = 23.06, *t*(15) = 1.83, *p* = 0.087, *d* = 0.46], and total advertising literacy [ALS-c: *M_pre_* = 93.69, *M_post_* = 94.19, *t*(15) = -0.211, *p* = 0.836, *d* = 0.05] were not significantly different across control sessions.

Lastly, there were no significant changes at the last control session compared to the first session in cognitive critical thoughts during commercials [*M_first_* = 1.13, *M_last_* = 1.31, *t*(15) = -0.25, *p* = 0.810, *d* = 0.06] and affective critical thoughts [*M_first_* = -0.44, *M_last_* = -0.25, *t*(15) = -0.35, *p* = 0.730, *d* = 0.09].

## Discussion

The goal of this pilot study was to determine whether brief, advertising literacy training could impact food choices in youth. To prevent (and treat) obesity from a developmental perspective, it is critical to help children rely less on taste in their food choices. Children’s cognitive processes are not yet fully developed to understand health attributes and their long-term benefits. The 1 week, four-session intervention significantly reduced the importance of “taste” in children’s food decisions. Children relied less on taste information when they made food choices after food advertising literacy training. However, the relative importance of taste attributes was not changed in the control condition. These findings demonstrated that the advertising literacy training was effective in reducing the taste importance in children’s food choices. This finding is remarkable when we consider the vast importance of the taste attribute in children. In children’s food choices, food taste is the predominant determinant of food choices, and health benefit is not considerably incorporated (Bruce et al., 2016), while the “healthiness” of the food makes a larger impact on adult food choices (Lim et al., 2018). Preferences for palatable, energy dense foods are quite common among children (Benton, 2004). In fact, preferences for sweet and salty tastes are predisposed responses that are quite persistent until adolescence (Cowart, 1981; Birch, 1999). Food taste has been regarded as the main factor determining food choices and consumptions (Glanz et al., 1998), and higher importance of food taste interferes with healthy and balanced diets (Vadiveloo et al., 2013). Reducing the importance of taste in food choices could contribute to the establishment of healthy eating habits (Raghunathan et al., 2006). These findings imply that strategies promoting less taste-oriented food choices could be helpful for childhood obesity prevention and intervention.

The brief training also influenced the children’s “self-talk” while viewing commercials, in that children engaged in more critical thought after the intervention. Findings from this study suggest that our advertising literacy training could be helpful for children to become more sensitive to advertising intent and tactics, and to process advertising information more critically. These findings imply that increased advertising literacy and healthy eating may enhance cognitive defenses against commercial-based external food cues. Although we know that advertising exerts a strong influence on children’s food choices and requests (Boyland et al., 2016), it is less clear how to *combat* these negative influences. It is challenging because food advertising could have multiple “levels” of effects, including the specific advertised brand, the more generic advertised category, and food overall (Buijzen et al., 2008). Some call for *less advertising exposure for children* (i.e., limit television, use DVR, limit screen time overall), while others believe that *improving advertising literacy* will yield the most benefits (Buijzen and Valkenburg, 2005).

The current findings must be interpreted with caution. Although results showed that improving advertising literacy would be beneficial in reducing the importance of taste in food decisions, our short intervention did not change children’s actual food decisions. This result may suggest that food taste still played a strong role in food choices, and the reduction was not enough to change children’s actual food choices. However, it is still noteworthy that the way or process of children’s food decisions was significantly changed (i.e., importance of taste on food decisions and critical thoughts). When considering childhood obesity intervention programs are usually designed for 1 to 2 h sessions for a few weeks to months (e.g., Sacher et al., 2010), a more intensive and lengthened training might have to be considered to extend the training effect to food decisions. Also, the training did not increase advertising literacy scores measured by ALS-c. It is possible that short exposures to narratives without further explanation about advertising literacy might not be enough to enhance advertising literacy levels significantly. Additionally, unlike our previous findings (Bruce et al., 2016), the exposure to food commercials did not increase the taste importance in the control condition in this study. It is uncertain, but the discrepancy might be related to differences in experimental paradigms between two studies. Whereas the previous study obtained food choices whiles watching food commercials in a blocked fashion through one-session experiment (Bruce et al., 2016), the current study obtained food choices after watching food commercials for 1 week. To better understand the effect of food commercials, future studies should examine how timing and duration of food commercial exposure systematically influences taste value computation in children’s food decisions.

Nevertheless, findings of our study showed that a brief advertising literacy training could be helpful for reducing dominant taste attributes in food choices. Also, advertising literacy training could be helpful for enhancing critical and skeptical thinking toward advertising. This effect was found when children had a chance to actively encode, process, and apply advertising literacy information through think aloud. Thus, parents and teachers may provide similar active opportunities to help their children more critically evaluate cues provided by food commercials. This may be especially important among children under the age of 6. Harris and Kalnova (2018) point out that younger children, particularly under the age of 6, are especially vulnerable to the effects of food advertising and the “self-regulation” done by the food industry itself is insufficient.

The study has several limitations. The sample size of this study was relatively small, so results should be extrapolated with caution. In addition, there was limited racial/ethnic diversity in the sample of children recruited. While we included the control group, this pilot study was not a randomized double-blind experiment. Studies need to not only examine attitudes toward advertising, food choices, but ultimately, ecologically valid food decisions. Although we tried to provide frequent training sessions over a week period, not surprisingly a training using short-video clips would not be enough to change actual food decision and self-reported advertising literacy levels. Future research should continue to investigate the utility of food advertising literacy training for the promotion of healthy eating and the prevention of childhood obesity.

## Ethics Statement

This study was carried out in accordance with the recommendations of Institutional Review Board at the University of Missouri-Kansas City, and the Human Subjects Committee at the University of Kansas Medical Center. The protocol was approved by the Human Subjects Committee at the University of Kansas Medical Center. All subjects gave written informed consent in accordance with the Declaration of Helsinki.

## Author Contributions

O-RH and AB contributed to the design of the experiment. O-RH, HK, and AB collected and analyzed the data. O-RH and AB prepared the manuscript. All authors contributed to the discussion of the results and implications and edited the manuscript.

## Conflict of Interest Statement

JB provides non-branded talks for Novartis, is a part-time employee of the National Hockey League, does consulting for Princeton University’s Department of Athletic Medicine, and is a consultant to Major League Soccer’s Sporting KC. The remaining authors declare that the research was conducted in the absence of any commercial or financial relationships that could be construed as a potential conflict of interest.
